# Supplementary material for: Divergence Analyses of Sperm DNA Methylomes between Monozygotic Twin AI Bulls
Source: Epigenomes. 2019 Sep 26;3(4):21. doi: 10.3390/epigenomes3040021 (PMC8594723; doi:10.3390/epigenomes3040021)
Supplement: Supplementary file 1 [file epigenomes-03-00021-s001.zip › epigenomes-554220-suppl-final/Supplementary_Figures.pdf]

Supplementary Figures

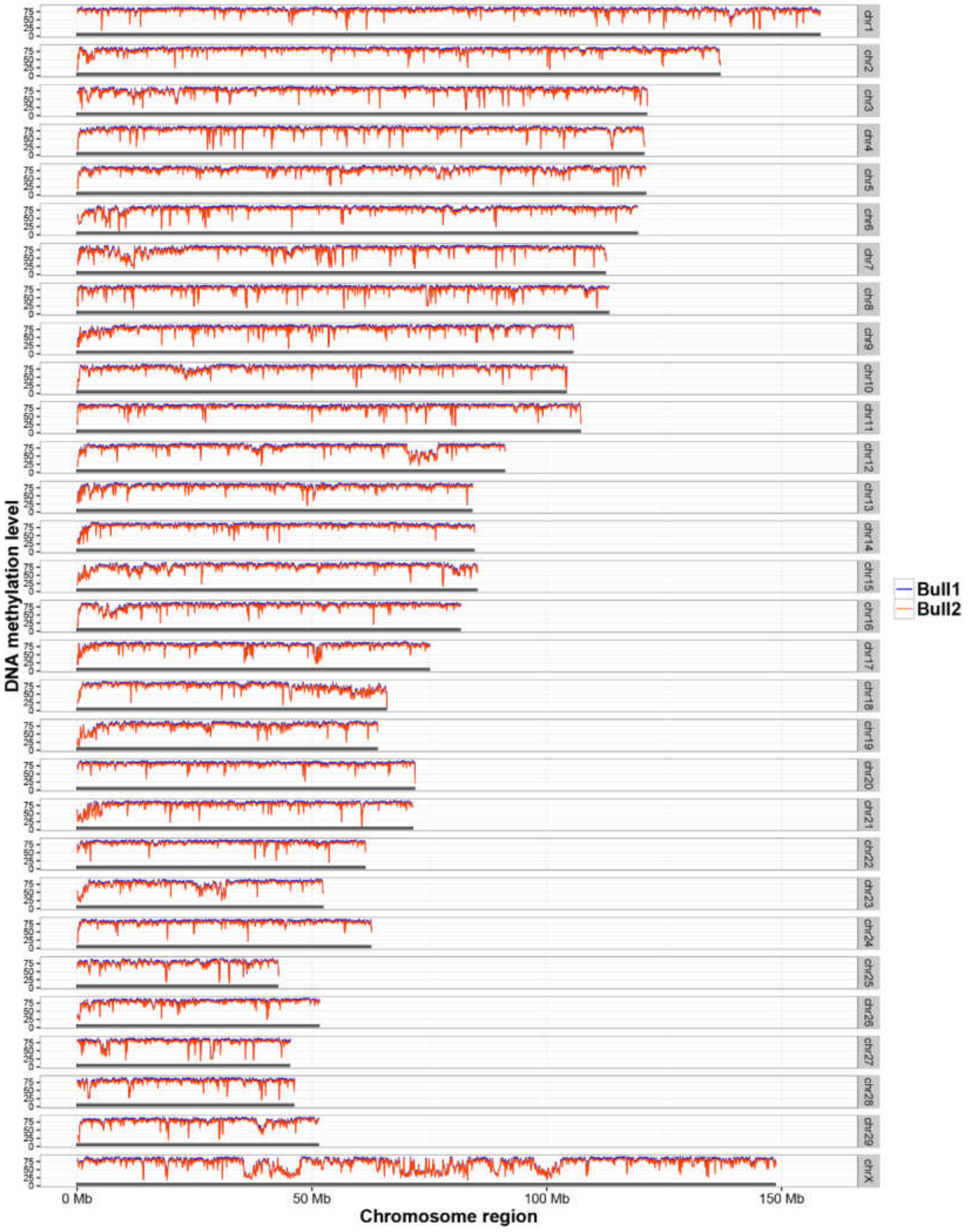

Supplementary Figure 1. A global view of CpG methylation levels along all chromosomes in sperm cells. The blue and red lines indicate CpG methylation levels of twin bulls in 100kb windows.

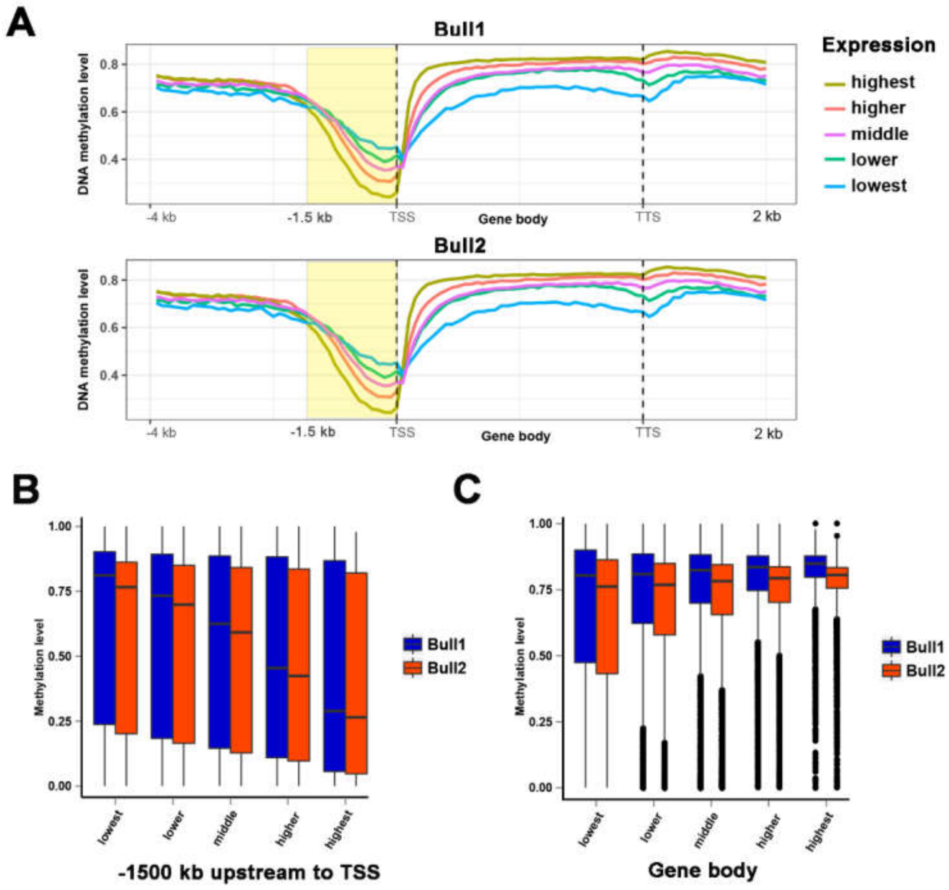

Supplementary Figure 2. The relationship between DNA methylation and gene expression in sperm cells of the MZ twin bulls. (A) DNA methylation levels around the TSS, gene body and TTS across five types of genes expression**.** Annotated genes were distributed into five categories based on their expression level: highest (n = 4923), higher (n = 4923), middle (n = 4923), lower (n = 4923) and lowest (n = 4924). The gene bodies, 4kb upstream and 2kb downstream were divided into 20 equally sized bins, respectively. DNA methylation level of each region is shown. **(B) (C)** Box plots showed average methylation degree of regions of −1500bp upstream to TSS and gene bodies in each gene expression categories.

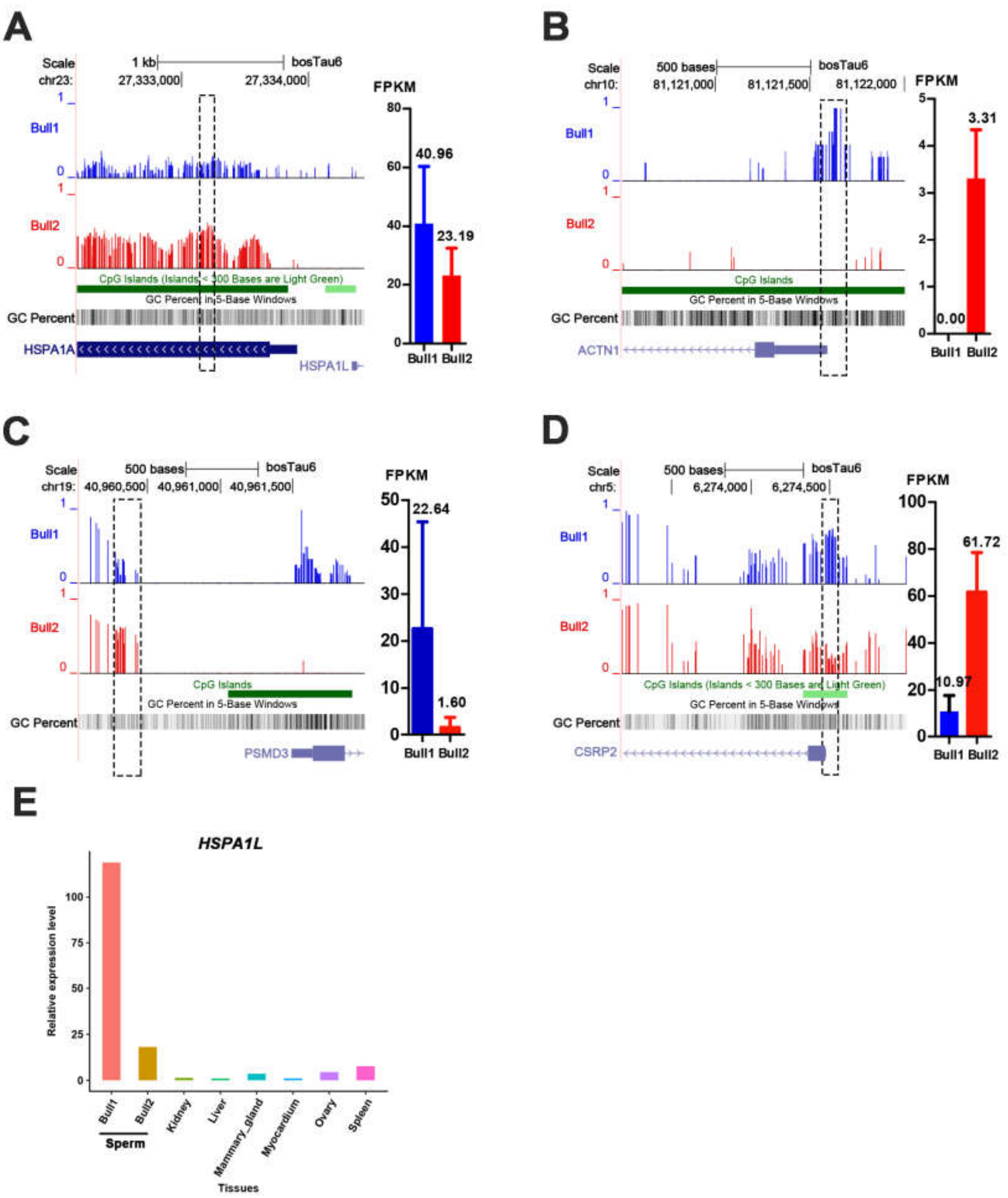

Supplementary Figure 3. Methylation levels as well as gene expression of four genes. The plots of methylation levels in the promoters of *HSPA1L* (A), *ACTN1* (B), *PSMD3* (C) and *CSRP2* (D) genes were prepared using UCSC Genome Browser. Blue bars and red bars (left) indicates the sperm methylation percentages of Bull1 and Bull2 (from 0 to 1, indicating 0% to 100% methylation), respectively. Each plot was prepared using the UCSC genome browser with the CpG islands and GC percent track. Bar plots (right) are the RNA expression in sperm samples of the twin bulls (labeled with FPKM values). **(E)** Relative expression levels of *HSPA1L* (measured by Quantitative Real-time PCR) in seven tissues including sperm cells of Bull1 and Bull2, kidney, liver, mammary gland, myocardium, ovary and spleen.
